# Gastro-Oesophageal Reflux Disease Outcomes Following Roux-en-Y Gastric Bypass Surgery in Patients with Obesity: A Systematic Review and Meta-analysis

**DOI:** 10.1007/s11695-025-07865-x

**Published:** 2025-04-24

**Authors:** Narek Sargsyan, Iihan Ali, Christopher Namgoong, Bibek Das, Matyas Fehervari, Michael G. Fadel

**Affiliations:** 1 Department of Surgery and Cancer, Imperial College London, London, UK; 2 Faculty of Medicine, Imperial College London, London, UK; 3 Department of General Surgery, Chelsea and Westminster Hospital, London, UK; 4 Department of Bariatric Surgery, Maidstone and Tunbridge Wells NHS Trust, Kent, UK

**Keywords:** Gastro-oesophageal reflux disease, Obesity, Bariatric surgery, Gastric bypass, Outcomes

## Abstract

We aimed to investigate the effect of primary Roux-en-Y gastric bypass (RYGB) on gastro-oesophageal reflux disease (GORD) in patients with obesity. A systematic review was performed using MEDLINE, Embase, Emcare, and CINAHL databases for studies that reported on GORD outcomes following RYGB for obesity (January 2000–November 2023). Fourteen studies with 28,027 patients underwent RYGB, with pooled analysis demonstrating a 47% (95% CI 34.0–59.0; *p* ≤ 0.005) improvement in GORD symptoms and 4.5% (95% CI 1.7–7.2; *p* ≤ 0.005) with worsening/new onset GORD. Postoperative DeMeester score improved by 16.49 points (95% CI 0.2–32.7; *p* ≤ 0.005) and 79.4% (95% CI 68.7–90.1; *p* = 0.01) completely discontinued proton-pump inhibitor therapy during the follow-up period. RYGB surgery may potentially improve GORD symptoms in patients with obesity, with an overall low incidence of de novo GORD.

**Supplementary Information:**

The online version contains supplementary material available at 10.1007/s11695-025-07865-x.

## Introduction

Obesity continues to be a significant global health concern that is growing in prevalence [1]. Obesity, defined as body mass index (BMI) > 30 kg/m^2^, is known to be associated with various co-morbidities including hypertension, hyperlipidaemia, type 2 diabetes mellitus, obstructive sleep apnoea, and cardiovascular disease. Gastro-oesophageal reflux disease (GORD) is a common condition observed in obesity potentially resulting in the increased prevalence of Barrett’s oesophagus and oesophageal adenocarcinoma, as well as a reduction in the quality of life of affected individuals [2]. Clinical history and questionnaire data are often insufficient to make a conclusive diagnosis of GORD in isolation. Conclusive evidence for GORD includes advanced grade erosive oesophagitis, long-segment Barrett’s mucosa or peptic strictures on oesophagogastro-duodenoscopy (OGD) or distal oesophageal acid exposure time > 6% on pH monitoring studies [3]. pH-impedance monitoring is considered to be the gold standard for the detection and characterisation of reflux episodes; however, it is expensive and not widely available [4].

In the management of obesity, bariatric surgery remains the only option for most patients when dietary interventions and pharmacotherapy fail. Roux-en-Y gastric bypass (RYGB) is an effective form of bariatric surgery, along with other procedures such as laparoscopic sleeve gastrectomy (LSG), that is well-established in treating obesity and reducing morbidity and mortality [5–8]. Although RYGB can be challenging from a surgical and anaesthetic point of view, it provides long-term effective weight loss and resolution of obesity-related co-morbidities [9, 10].

However, bariatric surgical treatment can itself exacerbate or result in GORD-related symptoms [11]. LSG has been shown to potentially have a higher risk of GORD; this incidence observed in literature may be a result of a specific patient subpopulation (selection bias) rather than the inherent property of LSG itself [12–14]. In terms of RYGB, the exact prevalence of GORD associated with RYGB remains uncertain. There is a lack of patient-specific data that can be used to guide discussions in a clinical setting regarding the direct short- and long-term improvements on GORD following RYGB, as well as the risk of developing de novo GORD symptoms. The likelihood of permanently stopping proton-pump inhibitors (PPI) following RYGB is also unclear.

The aim of this systematic review and meta-analysis is to directly assess the effect of primary RYGB on GORD in patients with obesity. This can in turn help with clinical decision-making and counselling in the management of patients with obesity and GORD. Outcomes including changes in GORD, PPI therapy, and DeMeester score following RYGB will be assessed. Perioperative outcomes, such as length of stay and changes in BMI, will also be presented.

## Methods

### Search Strategy

A literature search of MEDLINE, Embase, Emcare, and CINAHL databases was performed using the following Medical Subject Headings (MeSH) terms: gastro-oesophageal reflux disease, gastric reflux disease, acid reflux disease, reflux, Roux-en-Y, gastric bypass, laparoscopic gastric bypass, outcome, and efficacy. We retrieved articles published in the English language between 1st January 2000 and 1st November 2023 that reported GORD outcomes following RYGB. The reference lists from the selected studies were reviewed to identify any additional relevant studies. The full search strategy is presented in Supplementary Table S1.

The work has been reported in line with PRISMA (Preferred Reporting Items for Systematic Reviews and Meta-Analyses), and the study was registered in the PROSPERO database for systematic reviews [15].

### Study Selection and Data Extraction

All studies reporting on clinical outcomes of primary RYGB in the management of GORD were eligible for inclusion. Study types of randomised controlled trials (RCTs) and prospective and retrospective studies were included in this systematic review and meta-analysis. Articles were included if they presented data on GORD outcomes following RYGB which could be extracted (± comparison with other bariatric surgical procedures). The exclusion criteria were the following: (i) other specified forms of gastric bypass, such as single anastomosis sleeve ileal bypass (SASI) or one-anastomosis (Mini) gastric bypass (OAGB); (ii) articles published in a non-English language or in a book, (iii) letters to the editor, case reports, or conference abstracts; and (iv) animal studies.

Two authors conducted the search and identification independently against the inclusion and exclusion criteria, arriving at a final list of articles. Any disagreement was resolved by a third independent reviewer. The full-text of the articles were examined to determine ultimate inclusion in the final analysis. The following information was extracted from the manuscripts where reported: first author, year of publication, country, study design, demographics, use of PPI/H2 antagonists, DeMeester score baseline, and duration of follow-up. The preoperative investigations reported for GORD assessment were extracted (e.g. OGD, oesophageal manometry, impedance, or wireless pH study), along with the use of symptom questionnaires (e.g. GORD health-related quality of life (GORD-HRQL) [16]). Perioperative outcomes extracted include operative time, GORD-HRQL score, re-operation, and mortality rate. The primary outcomes extracted include improvement in GORD symptoms reported by patients, no improvement in GORD symptoms, new or worsening GORD symptoms, discontinuation of PPI therapy, and preoperative versus postoperative DeMeester score. Secondary outcomes extracted include length of stay and change in BMI post-RYGB.

### Quality Assessment

The quality of all observational studies was assessed using the Newcastle–Ottawa Scale (NOS) [17] by examining three factors: (i) method of patient selection, (ii) comparability of the study groups, and (iii) number of outcomes reported. The full score was nine stars, and studies that had a score of seven stars or more were deemed high quality. All studies were rated independently by two authors, with any differences resolved by consensus. The strength of clinical data and subsequent recommendations were also graded according to the Oxford Centre for Evidence-Based Medicine [18]. Levels of evidence were as follows: level 1, properly powered and conducted RCT or systematic review with meta-analysis; level 2, well-designed controlled trial without randomisation or prospective comparative cohort trial; level 3, case–control study or retrospective cohort study; and level 4, case series or cross-sectional study.

### Statistical Analysis

The weighted mean difference (WMD) was calculated, and the logarithm of DerSimonian-Laird (DL) with 95% confidence intervals (CI) was used as the primary summary statistic. A *p* value of < 0.05 was deemed to be statistically significant. Between-study heterogeneity was assessed using the *I*^2^ value to measure the degree of variation not attributable to chance alone. This was graded as low (*I*^2^ < 25%), moderate (*I*^2^ = 25–75%), or high (*I*^2^ > 75%) and *p* values of ≤ 0.05 were taken to indicate statistical significance. Meta-analysis of data was conducted using a random-effects model, and all statistical analyses including the forest plots were performed using STATA version 16.

## Results

### Study Selection and Patient Characteristics

**Fig. 1:**
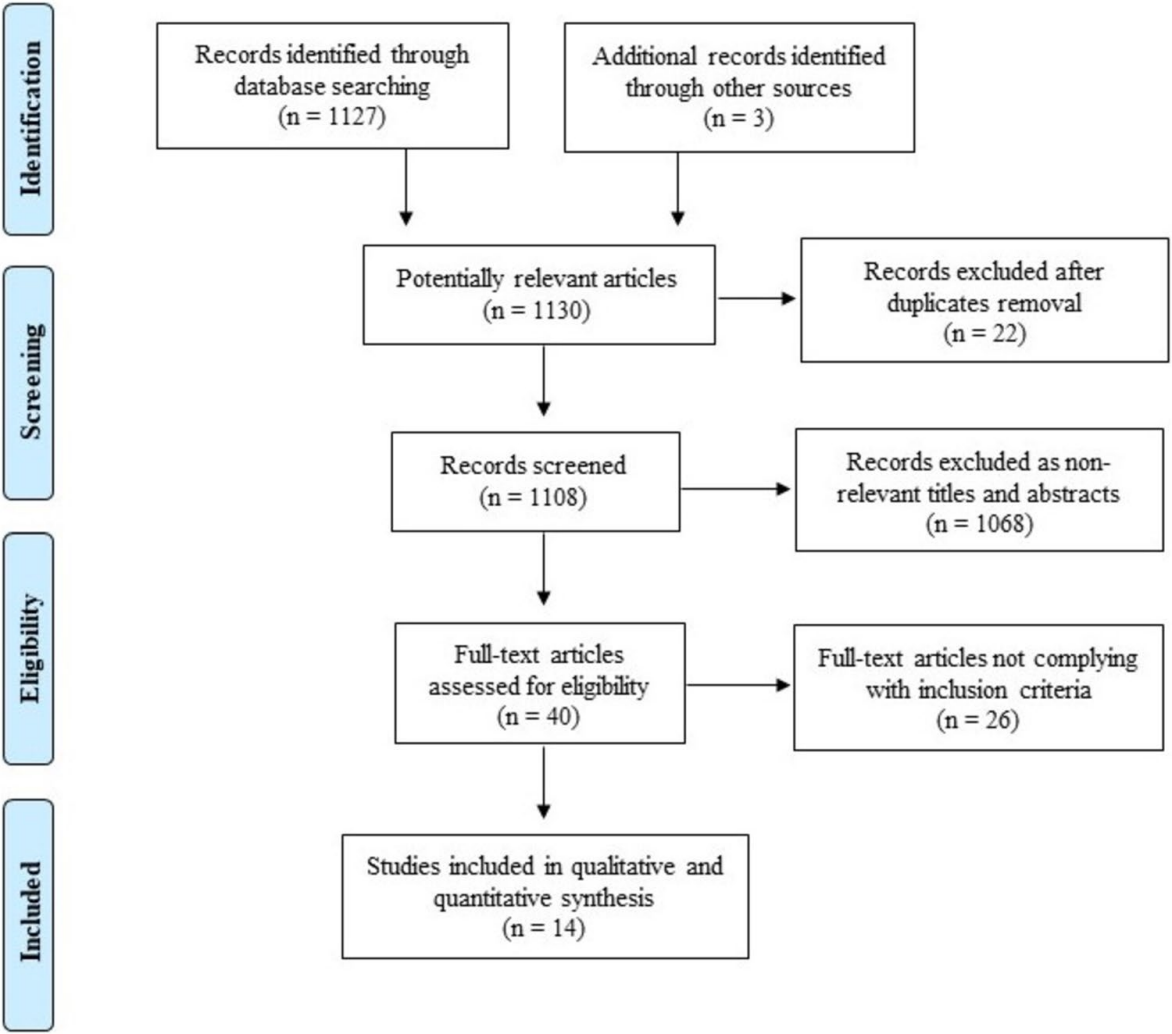
The flowchart shows the literature search and study selection process according to the PRISMA guidelines

The initial database search and additional records identified 1130 publications. A total of 1090 articles were excluded after title and abstract review and removal of duplicates. Forty articles were fully reviewed, and 14 studies [2, 19–31] were included in the qualitative and quantitative analysis. A total of 28,027 bariatric patients underwent a gastric bypass procedure. The PRISMA diagram of the literature search is displayed in Fig. 1.

The study and patient characteristics, level of evidence, and quality assessment of the included studies are detailed in Table 1. Six studies were from the USA, with the remaining studies from Italy, Brazil, France, Mexico, Germany, Spain, and Sweden. Eight studies were prospective studies, five studies were retrospective studies, and there was one RCT found. The NOS was applied to the 13 observational studies, with 10 studies being deemed to be of high quality (Supplementary Table S2). Six studies reported outcomes on RYGB only, while eight studies presented outcomes of RYGB compared to other bariatric or anti-reflux procedures such as laparoscopic sleeve gastrectomy (LSG), laparoscopic adjustable gastric band, or laparoscopic Nissen’s fundoplication.

**Table 1:** Study design and baseline demographics of studies included in systematic review and meta-analysis. *GORD*, gastro-oesophageal reflux disease; *LAGB*, laparoscopic adjustable gastric band; *LNF*, laparoscopic Nissen’s fundoplication; *LSG*, laparoscopic sleeve gastrectomy; *NOS*, Newcastle–Ottawa Scale; *OGD*, oesophagogastro-duodenoscopy; *PPI*, proton-pump inhibitor; *RYGB*, Roux-en-Y gastric bypass; *SD*, standard deviation; *VBG*, vertical banded gastroplasty; — = not reported; * = studies deemed to be high quality according to NOS

Author, year	Country	Study design (level of evidence)	Type of bariatric procedure	Sample size	Mean/median age ± SD, years (range)	Sex, males (%)	Mean/total follow-up period, months/years	Mean/median BMI ± SD (range), kg/m^2^	PPI/H2 antagonists, *n* (%)	Preoperative investigations performed for GORD assessment (*n*; %)	DeMeester score baseline ± SD (range)	NOS
Santonicola et al. [19], 2022	Italy	Retrospective (3)	RYGB	45	40.4 ± 10.4	10 (22.2)	99.9 months	42.1 ± 5.4	–	–	–	7*
Leslie et al. [20], 2021	USA	Retrospective (3)	RYGB and VSG	16,994 (8362 RYGB and 8362 LSG)	46.8 ± 10.8	1725 (20.6)	4.1 years	-	1013 (12.1)	OGD (*n* = 3746; 44.8); oesophageal manometry (*n* = 99; 1.2) and pH monitoring study (*n* = 59; 0.7)	-	9*
Holmberg et al. [21], 2019	Sweden	Retrospective (3)	RYGB	2454	46.1 ± 9.8	449 (18.3)	4.6 years	–	–	–	–z	9*
Merrouche et al. [22], 2007	France	Prospective (2)	RYGB and LAGB	27 (15 RYGB and 12 AGB)	38.4 ± 10.9	-	-	48.6 ± 5.7	-	In all patients: OGD; oesophageal manometry and 24-h pH monitoring study	24.8 ± 13.7	7*
Navarini et al. [23], 2020	Brazil	RCT (1)	RYGB and LSG	75 (40 RYGB and 35 LSG)	39 ± 12.1	8 (20)	12 months	42.7 ± 5.7	–	In all patients: OGD; oesophageal manometry and 24-h pH monitoring study; barium swallow X-ray	10.8 (5–29.2)	8*
Pallati et al. [2], 2013	USA	Prospective (2)	RYGB, LAGB and LSG	22,870 (14,078 RYGB)	47.5 ± 10.9	2520 (17.9)	6 months	47.1 ± 8.18	–	–	–	8*
Korenkov et al. [24], 2002	Germany	Prospective (2)	RYGB and LAGB	53 (20 RYGB and 30 LAGB)	34 (33–54)	2 (10)	22 months	54 ± 10.7		OGD and oesophageal manometry in all patients; 24-h pH monitoring study (not specified)	66.5 ± 35.4	7*
Gilmore et al. [25], 2013	USA	Retrospective (3)	RYGB	1047	49.6 ± 8	187 (17.8)	6 months	36.7	–	–	–	7*
Ortega et al. [26], 2004	Spain	Prospective (2)	RYGB and VBG	50 (40 RYGB and 10 VBG)	36.0 ± 7.5	9 (22.5)	12 months	54.5 ± 8.4	-	In all patients: oesophageal manometry and 24-h pH monitoring study; isotopic oesophageal emptying	18.9 ± 15	6
Patterson et al. [27], 2003	USA	Prospective (2)	RYGB and LNF	12 (6 RYGB and 6 LNF)	39.8	–	14.6 months	55 ± 20.7	6 (100)	In all patients: OGD; oesophageal manometry and 24-h pH monitoring study	34.76 ± 53.6	8*
Rebecchi et al. [28], 2016	Italy	Prospective (2)	RYGB	72 (26 with preop GORD)	40.1 ± 10.5	5 (6.9)	60 months	44.4 ± 3.9	32 (44.4)	In all patients: OGD; oesophageal manometry and 24-h pH monitoring study	-	8*
Perry et al. [29], 2004	USA	Prospective (2)	RYGB	57	43 (22–67)	6 (10.5)	18 months	43 (35–67)	54 (94.7)	None	–	6
Ehlers et al. [30], 2022	USA	Retrospective (3)	RYGB and LSG	10,451 (1771 RYGB and 8680 SG)	46.5	363 (20.5)	12 months	48.53	–	None	–	9*
Mejia-Rivas et al. [31], 2008	Mexico	Prospective (2)	RYGB	20	38.9 ± 6.9	4 (20)	6 months	48.5 ± 6.2	–	In all patients: oesophageal manometry and 24-h pH monitoring study	48.3 ± 8.5	6

The mean/median age of patients ranged from 34.0 to 49.6 years and the mean/median BMI ranged from 36.7 to 55.0 kg/m^2^. The mean/total follow-up period ranged from 6 to 100 months. Eight out of the 14 studies [20, 22–24, 26–31] reported the specific preoperative investigations performed for GORD assessment prior to RYGB. These investigations included oesophageal manometry and pH monitoring study (*n* = 8 studies), OGD (*n* = 6), barium swallow X-ray (*n* = 1), and isotopic oeosphageal emptying (*n* = 1). Five studies [23, 28–31] reported using a symptom questionnaire including GORD-HRQL (*n* = 3), Short Form 36 (SF- 36) health survey questionnaire [32] (*n* = 1), Carlsson-Dent Questionnaire (CDQ) [33] (*n* = 1), and a validated symptom questionnaire for GORD [34] (*n* = 1).

### Impact on GORD

**Fig. 2:**
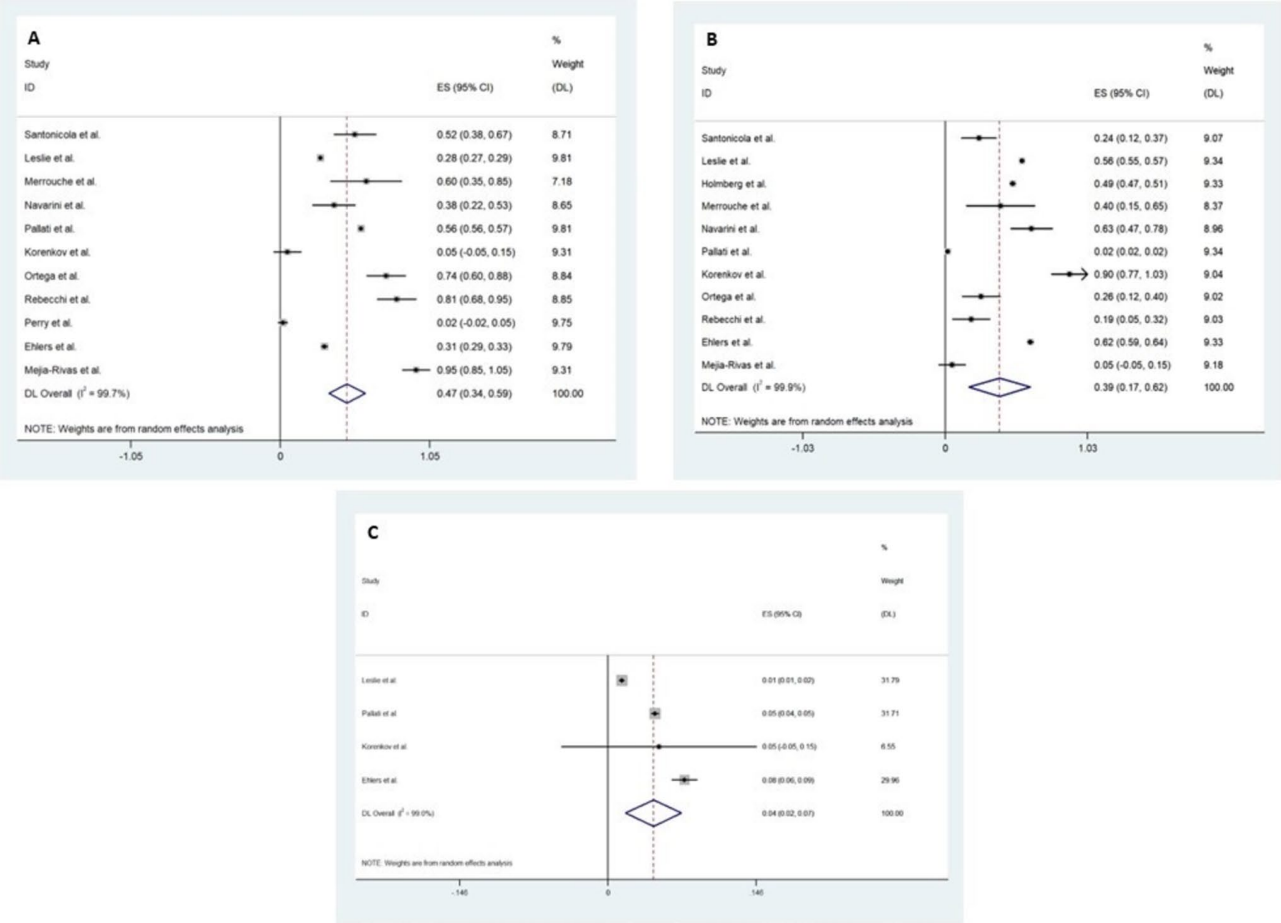
Forest plot demonstrating **A** improvement of GORD symptoms, **B** no improvement of GORD symptoms, and **C** new or worsening GORD symptoms following Roux-en-Y gastric bypass

Eleven studies, a total of 24,520 patients, reported on GORD symptoms, with the pooled analysis revealing a 47.0% (95% CI 34.0–59.0; *p* ≤ 0.005; *I*^2^ = 99.7) remission or improvement of GORD symptoms (Fig. 2A). In a pooled analysis of eleven studies, including 26,917 patients, 39.5% (95% CI 17.2–61.7; *p* ≤ 0.005; *I*^2^ = 99.9) reported no improvement of GORD symptoms post-RYGB surgery (Fig. 2B). Pooled analysis of four studies, including 24,231 patients, demonstrated 4.5% (95% CI 1.7–7.2; *p* ≤ 0.005; *I*^2^ = 99.0) had new or worsening GORD symptoms following RYGB (Fig. 2C).

### Impact on PPI Discontinuation and DeMeester Score

**Fig. 3:**
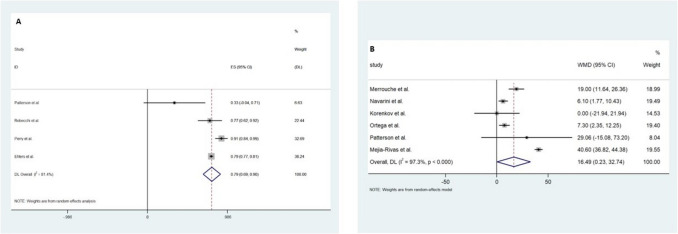
Forest plot demonstrating **A** discontinuation of proton-pump inhibitors following Roux-en-Y gastric bypass and **B** preoperative versus postoperative DeMeester score

Four studies, a total of 1906 patients, demonstrated that 79.4% (95% CI 68.7–90.1; *p* = 0.01; *I*^2^ = 81.4) discontinued using PPIs post-RYGB due to improvement/resolution of GORD symptoms (Fig. 3A). Weighted mean analysis of six studies, including 141 patients, revealed a 16.49 (95% CI 0.23–32.74; *p* = 0.047; *I*^2^ = 97.3) improvement of DeMeester score after RYGB (Fig. 3B). A preoperative GORD-HRQL score of 33.8 ± 8.2 was reported by one study [28]. The postoperative GORD-HRQL score was reported by two studies [28, 29], which was found to have a mean of 12.1 ± 14.2.

### Perioperative Outcomes and Changes in BMI

**Fig. 4:**
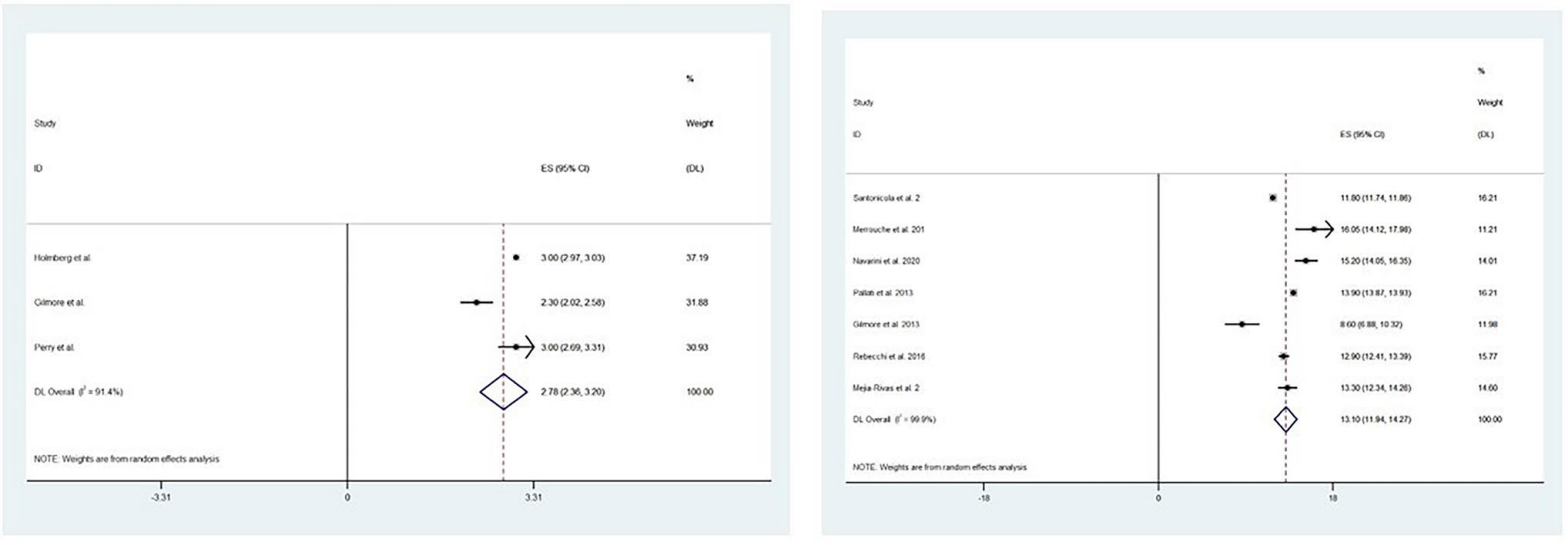
Forest plot demonstrating **A** length of stay and **B** changes in BMI following Roux-en-Y gastric bypass

The mean operative time for RYGB was 151.3 min. Pooled analysis of three studies, including 3558 patients, showed a mean length of hospital stay of 2.7 days (95% CI 2.3–3.2; *p* ≤ 0.005; *I*^2^ = 91.4) after RYGB surgery (Fig. 4A). Three [21, 28, 29] and two studies [29, 30] reported on re-operation rate and endoscopic dilatation which was 2.0% and 6.2% on average, respectively. Seven studies reported on mortality rate during the follow-up period. Only one study [21] had mortality within 90 days following RYGB, which was reported as 0.2% (cause of death was not stated). In terms of weight loss, seven studies of 15,317 patients reported on pre- and post-RYGB change in BMI. Pooled analysis of these studies demonstrated a 13.10 kg/m^2^ (95% CI 11.94–14.27; *p* ≤ 0.005; *I*^2^ = 99.86) reduction in BMI (Fig. 4B).

## Discussion

Obesity is a well-established risk factor for GORD. In the severely obese population, bariatric surgery has interestingly been reported as both the cure and the cause of GORD symptoms [11, 12]. RYGB is one of the most common bariatric procedures and is considered the procedure of choice to treat patients with severe obesity and GORD. Although, several studies have demonstrated excellent outcomes for weight loss and improvement of GORD, some authors have reported the new onset or recurrence of GORD [19].

To our knowledge, this study provides the largest systematic review and meta-analysis (28,027 patients across 14 studies) that specifically evaluated GORD outcomes following RYGB in patients with obesity. This study demonstrated a 47.0% remission of GORD symptoms, with 39.5% not reporting any changes (worsening nor improvement) of their GORD symptoms after undergoing RYGB. The other key findings of this study include an overall DeMeester score improvement of 16.49 points, with 79.4% discontinuing PPIs due to an improvement or resolution of GORD symptoms during the follow-up period. Only 4.5% reported worsening or new onset of GORD symptoms post-RYGB. Furthermore, we found that not all studies routinely performed or reported preoperative investigations (including the gold standard pH monitoring study) as part of their GORD assessment. There was also variability in the use of questionnaires for GORD assessment in this review, with currently a total of approximately 40 symptom questionnaires reported in the literature [35].

Our study therefore suggests that RYGB may potentially improve reflux disease in patients with obesity. This finding shares similarities with other studies in the literature. Gu et al. performed a meta-analysis to investigate the relationship between bariatric surgery and GORD in a pooled patient cohort of 2537 patients who underwent RYGB and 2563 patients had LSG [36]. Their analysis showed that RYGB was more effective for treating GORD than LSG and the incidence of newly onset GORD after RYGB was also lower. In addition, Elzouki et al. [37] conducted a dose–response meta-analysis (4715 LSG and 580 RYGB patients) to find that 63.4% (95% CI 32.5–72.2) experienced an improvement in GORD symptoms. The authors concluded that bariatric surgery may improve GORD symptoms in patients with obesity who underwent LSG; however, the most favourable effect is likely to be found with RYGB. Suter [38] performed a narrative review of the interactions between obesity, GORD, and RYGB, suggesting that RYGB may have anti-reflux properties by reducing oesophageal acid exposure and by improving hypercontractility of the oesophagus. RYGB may also lead to a decrease in PPIs use due to a marked reduction of acid secretion in the gastric pouch. The review concluded that RYGB has overall positive effects on GORD and could be seen as the procedure of choice for patients with GORD who are candidates for bariatric surgery. However, the review also noted that RYGB does not lead to GORD remission in all patients and may even cause GORD in a minority of patients.

There are multiple mechanisms postulated that predispose patients with obesity to reflux including transient lower oesophageal sphincter relaxations thus leading to increased acid exposure and GORD. There is also a strong association between abdominal diameter and reflux symptoms [39]. Patients with obesity are more likely to have increased intragastric pressure and disruption of oesophagogastric junction which predisposes them to reflux. A higher incidence of hiatus hernias has been observed in obesity, which can lead to functional impairment of the gastroesophageal junction which plays an important role in preventing reflux [40, 41]. The compromise in the oesopagogastric junction and hiatus hernia, together with a rise in intragastric pressure from abdominal obesity, leads to the development of GORD. Other studies have observed a link between adiponectin and leptin from adipocytes in obesity and reflux, which is thought to be from the dysregulation of the levels of these proteins in obesity [42]. However, further studies are required in this field to understand this link in obesity and reflux.

In total, GORD has been reported in up to 73% of bariatric surgery candidates [43]. RCTs have reported high rates of GORD improvement following RYGB compared to other bariatric procedures such as LSG [36]. This may be related to higher weight loss achieved with RYGB and pathophysiological differences including reduction of parietal cells responsible for gastric acid secretion. Concomitant hiatus hernia repair during bariatric procedure may lead to improvement in GORD [44–48]. De novo reflux following bariatric surgery has emerged as a topic of concern as the incidence of bariatric surgery rises. The Swiss Multicenter Bypass or Sleeve Study (SM-BOSS) trial reported lower incidence of de novo reflux following RYGB in comparison to sleeve gastrectomy [49]. Revisional bariatric surgery to RYGB has also been reported to be an effective treatment in patients with refractory reflux following mini-gastric bypass [50]. Several pathophysiological mechanisms for developing de novo or worsening GORD following RYGB have been proposed, including anastomotic stenosis, large gastric remnant, gastrogastric fistula, and functional impairment of the gastroesophageal junction. Retained gastric antrum syndrome following RYGB can lead to hypergastrinaemia as the gastrin cells in the retained antral tissue are not exposed to luminal acid, resulting in continuous secretion of gastrin and intense stimulation of parietal cells producing high levels of acid, leading to GORD and marginal ulceration [51].

Following bariatric surgery, several lifestyle modifications are recommended to reduce the risk of GORD. This includes gradual advancing of diet from liquids to smaller portions of healthy food as well as avoidance of factors that exacerbate reflux such as alcohol, smoking, caffeine, chocolate, and postprandial supination. Overfilling the stomach with large calorie heavy meals can lead to delayed emptying and reflux. Novel potassium-competitive acid blockers, as well as PPIs, have been reported to provide benefit in the management of GORD [52, 53].

In addition, postoperative complications such as stenosis, angulation, and marginal ulceration should be recognised and treated appropriately, as these can lead to raised intragastric pressure and GORD [54]. Gastro-jejunal strictures following RYGB leading to reflux can also be managed with endoscopic balloon dilatation [55]. Gastrogastric fistulas can lead to weight gain and GORD, and various endoscopic repair techniques have been utilised as part of the management [56]. Another important factor and critical in decreasing the risk of GORD following RYGB is the surgical technique in creating optimal gastric pouch of 20–30 cc in volume involving the lesser curve of the stomach and average limb length of 120 cm [57, 58]. The Hill procedure using the phrenoesophageal bundle for persistent GORD following RYGB has been reported to be a treatment option in a small number of centres [59]. The use of magnetic sphincter augmentation devices, such as the LINX system, has also shown promising results in treatment of GORD following bariatric surgery, although further data and RCTs are required to establish the precise long-term safety and efficacy of this approach [60–62].

### Strengths and Limitations

The strength of this systematic review and meta-analysis is that it included a large sample size of 28,027 patients that had GORD outcomes presented following RYGB. However, this study is subject to limitations that should be mentioned. There was significant between-study heterogeneity found which may be due to the variation in eligibility criteria of studies and patient demographics. The majority of the studies were prospective or retrospective, with only one RCT identified, and not all studies assessed every aspect reviewed by this meta-analysis. Five studies [2, 20, 21, 25, 30] in particular contributed to the large sample size in this meta-analysis. Not all studies in this review presented the GORD assessment or diagnostic methods, and very few studies reported on baseline co-morbidities related to obesity and sequalae of GORD and therefore this was not presented in our systematic review and meta-analysis. There was also a significant variation found in the length of follow-up between the included studies, ranging from 6 to 100 months. Of note, a prospective RCT with pre-operative pH/manometry randomised to PPI versus surgery would be needed to better understand the outcomes.

## Conclusions

The findings of this systematic review and meta-analysis demonstrated that RYGB has potentially favourable outcomes in the improvement of GORD in patients with obesity, with a suggestive low incidence of de novo GORD symptoms. This may have important implications for counselling patients with GORD prior to bariatric surgery. Further RCTs are required that directly compare RYGB with other bariatric procedures to confirm the long-term impact and resolution of GORD symptoms.

## Supplementary Information

Below is the link to the electronic supplementary material.

Supplementary file1 (DOCX 31 KB)

## Data Availability

No datasets were generated or analysed during the current study.
