# Supplementary material for: Gastro-Oesophageal Reflux Disease Outcomes Following Roux-en-Y Gastric Bypass Surgery in Patients with Obesity: A Systematic Review and Meta-analysis
Source: Obes Surg. 2025 Apr 24;35(6):2321–32. doi: 10.1007/s11695-025-07865-x (PMC12130072; doi:10.1007/s11695-025-07865-x)
Supplement: Supplementary file 1 — Supplementary file1 (DOCX 31 KB) [file 11695_2025_7865_MOESM1_ESM.docx]

**Supplementary material**

| **Search** | **Query** | **Results** |
| --- | --- | --- |
| 1 | (bariatric surg* or weight loss surg* or weight reduction surg* or obesity surg*) | 26799 |
| 2 | ((weight loss or weight reduction or obes* or bariatric*) adj3 surg*) | 30611 |
| 3 | (gastric bypass or "sleeve gastrectomy with duodenal switch" or implantable gastric stimulation or "endoscopic sleeve gastroplasty") | 17135 |
| 4 | exp Bariatric Surgery/ | 32595 |
| 5 | 1 or 2 or 3 or 4 or 5 or 6 | 47622 |
| 6 | (Gastroesophageal reflux disease or GERD) | 16180 |
| 7 | (gastro-oesophageal reflux disease or GORD) | 2597 |
| 8 | (gastric reflux disease or acid reflux disease or reflux or gastroesophageal reflux) | 69108 |
| 9 | exp Gastroesophageal Reflux/ | 28945 |
| 10 | 6 or 7 or 8 or 9 | 69881 |
| 11 | 5 and 10 | 2105 |
| 12 | limit 11 to (english language and yr="2000 -Current") | 1906 |
| 13 | 12 and "Case Reports".sa_pubt. | 151 |
| 14 | 12 and "Letter".sa_pubt. | 88 |
| 15 | 12 and "Consensus Development Conference".sa_pubt. | 4 |
| 16 | 12 and "Editorial".sa_pubt. | 40 |
| 17 | 12 and "Guideline".sa_pubt. | 1 |
| 18 | 12 and "Practice Guideline".sa_pubt. | 6 |
| 19 | 12 and "Research Support, N.I.H., Extramural".sa_pubt. | 18 |
| 20 | 13 or 14 or 15 or 16 or 17 or 18 or 19 | 299 |
| 21 | 12 not 20 | 1607 |
| 22 | (roux-en-y or gastric bypass roux-en-y or roux-en-y laparoscopic gastric bypass or "RYGB Proximal" or "RYGB Distal" or RYGB) | 15319 |
| 23 | Gastric Bypass/ | 12144 |
| 24 | 22 or 23 | 20122 |
| 25 | 10 and 24 | 1677 |
| 26 | limit 25 to (english language and yr="2000 -Current") | 1224 |
| 27 | 26 and "Case Reports".sa_pubt. | 135 |
| 28 | 26 and "Research Support, Non-U.S. Gov't".sa_pubt. | 89 |
| 29 | 26 and "Letter".sa_pubt. | 42 |
| 30 | 26 and "Comment".sa_pubt. | 45 |
| 31 | 26 and "Research Support, N.I.H., Extramural".sa_pubt. | 7 |
| 32 | 26 and "Video-Audio Media".sa_pubt. | 19 |
| 33 | 27 or 28 or 29 or 30 or 31 or 32 | 279 |
| 34 | (outcome* or effective* or efficacy) | 5521518 |
| 35 | patient outcome assessment/ or patient reported outcome measures/ or treatment outcome/ | 1151442 |
| 36 | 34 or 35 | 5521518 |
| 37 | 25 and 36 | 1188 |
| 38 | limit 37 to (english language and yr="2000 -Current") | 1127 |

**Table S1.** Systematic search strategy created for MEDLINE, Embase, Emcare and CINAHL databases from 1^st^ January 2000 to 1^st^ November 2023.

| **Author, Year** | **Selection** | | | | **Comparability** | | **Outcome** | | | **Total score** |
| --- | --- | --- | --- | --- | --- | --- | --- | --- | --- | --- |
|  | **Representativeness of the exposed cohort** | **Selection of the non- exposed cohort** | **Ascertainment of exposure** | **Outcome not resent at start** | **Comparability of cohorts on the basis of the design or analysis** | **Study controls for additional factor** | **Assessment of outcome** | **Was follow-up long enough** | **Adequacy of follow up** |  |
| Santonicola et al. [19], 2022 | ✩ |  | ✩ | ✩ | ✩ |  | ✩ | ✩ | ✩ | 7 |
| Leslie et al. [20], 2021 | ✩ | ✩ | ✩ | ✩ | ✩ | ✩ | ✩ | ✩ | ✩ | 9 |
| Holmberg et al. [21], 2019 | ✩ | ✩ | ✩ | ✩ | ✩ | ✩ | ✩ | ✩ | ✩ | 9 |
| Merrouche et al. [22], 2007 | ✩ |  | ✩ | ✩ | ✩ |  | ✩ | ✩ | ✩ | 7 |
| Navarini et al. [23], 2020 | ✩ | ✩ | ✩ | ✩ | ✩ | ✩ | ✩ | ✩ |  | 8 |
| Pallati et al. [2], 2013 | ✩ | ✩ | ✩ | ✩ | ✩ | ✩ | ✩ | ✩ |  | 8 |
| Korenkov et al. [24], 2002 | ✩ | ✩ | ✩ | ✩ | ✩ | ✩ | ✩ |  |  | 7 |
| Gilmore et al. [25], 2013 | ✩ | ✩ | ✩ | ✩ | ✩ | ✩ | ✩ |  |  | 7 |
| Ortega et al. [26], 2004 | ✩ |  | ✩ | ✩ | ✩ | ✩ | ✩ |  |  | 6 |
| Patterson et al. [27], 2003 | ✩ | ✩ | ✩ | ✩ | ✩ | ✩ | ✩ |  | ✩ | 8 |
| Rebecchi et al. [28], 2016 | ✩ | ✩ | ✩ | ✩ | ✩ | ✩ | ✩ | ✩ |  | 8 |
| Perry et al. [29], 2004 | ✩ |  | ✩ | ✩ | ✩ |  | ✩ |  | ✩ | 6 |
| Ehlers et al. [30], 2022 | ✩ | ✩ | ✩ | ✩ | ✩ | ✩ | ✩ | ✩ | ✩ | 9 |
| Mejia-Rivas et al. [31], 2008 | ✩ |  | ✩ | ✩ | ✩ |  | ✩ |  | ✩ | 6 |

**Table S2.** Newcastle-Ottawa Scale scoring for observational studies included in systematic review.
